# Uses for the Oxysulphate of Zinc

**Published:** 1892-12

**Authors:** 


### SELECTIONS.

### Uses for the Oxysulphate of Zinc.

Dr. W. D. Miller, of Berlin, says: "The oxysulphate of zinc is a material which is not as extensively used as it deserves to be."

The preparation he uses is Fletcher's Artificial Dentine. He adds:

"The one I use consists of a white or yellowish-white powder, oxide of zinc, and a syrupy, opaque liquid, whose exact composition I am unable to give. As I have been informed by the manufacturer, 'the artificial dentine is an oxysulphate in the same sense that the oxychlorides are oxychlorides; the hydrochloric acid in the basic compound is replaced by sulphuric acid, and it is really a basic sulphate of zinc with a small proportion of free oxide.'

"When mixed moderately thick it hardens quite rapidly, in fact, as soon as it is in the cavity it is hard enough to undergo the necessary trimming. The time required for its setting can, however, be increased *ad libitum* by mixing it sufficiently thin. Like other preparations of its kind, it rapidly deteriorates in quality if any impurities obtain access to it, or if the bottles are not kept perfectly corked.

"When fully hardened it has not quite the hardness of plaster of Paris, but is a little tougher. In positions where it is not affected by mastication, I have known it to last as long as two years, though it is solely for temporary purposes that I use or recommend it. It is practically non-irritant; a quantity of the material mixed, being taken upon the tongue, produces about the sensation of a half per cent. solution of carbolic acid.

"1. For capping exposed pulps. When the pulp has been fully prepared for capping I mix a small quantity of the cement to such a consistency that when it is taken upon the point of an excavator it does not flow off from it, but still is sufficiently thin to hang down in the shape of a minute drop. If a drop of cement of this consistency a little larger than a pin head is brought into contact with the point of exposure, it spreads itself out over the surface of the pulp, adapting itself perfectly to its irregularities and forming a much more perfect covering than can be obtained with asbestos, pieces of paper, gutta percha or any other material which cannot be applied in a semi-fluid state; besides, what is of greatest importance, it may be applied without a trace of pressure.

"An antiseptic capping may easily be produced by incorporating the antiseptic into the capping material, though some substances interfere with the hardening. As soon as the cap has hardened (about two minutes more if the cement was mixed very thin), the filling may be completed. If it is a doubtful case, I finish the operation with *oxysulphate* and wait three or four weeks. If it is a fresh exposure and the pulp healthy, I finish with *oxyphosphate.* If finally I have every reason to exclude the possibility of a failure, I place a layer of oxyphosphate, over the cap of oxysulphate, and complete the operation with a permanent filling material at once. The directions for use accompanying the material appear to me to be fundamentally wrong; my manner of using it will, I am sure, give better results.

"2. In the operation of perforating or removing hard fillings from pericementitic teeth, I have found the oxysulphate to be of the greatest service. How painful if not unbearable for the patient, and how trying to the operator it is to operate upon a tooth which may be so sensitive that the slightest touch causes excruciating pain, we all know, and yet this operation may be made almost or quite painless. Dry the tooth to be operated upon as well as the adjoining tooth, on each side, with bibulous paper, then mix a large quantity of the oxysulphate, say half a thimble full, and plaster it with a broad spatula upon the lingual as well as labial surface of the three teeth, slightly pressing upon it so as to force it between the teeth. It hardens sufficiently in one or two minutes to fix the tooth immovably between the adjoining teeth. The ease with which the operation of removing the filling may then be performed is often a matter of surprise both to patient and operator. In these cases plaster of Paris may take the place of oxysulphate.

"3. In like manner oxysulphate or plaster of Paris may be used during the operation of filling with gold, for fixing teeth which have become loosened, no matter by what process.

"4. I also sometimes make use of the oxysulphate for pressing the gums away from the cervical margin of cavities, particularly in wedgeshaped cavities where cotton cannot be made to hold. Dry the cavity thoroughly and fill it with cement mixed rather thick, and when it has begun to harden press upon it with a pledget of cotton. The cement spreads out and forces the gums back at the margin of the cavity.

"5. For enclosing applications on cotton of whatever nature I have found the oxysulphate vastly superior to gutta percha. Whether I have to make an application to an inflamed pulp, for the purpose of sterilizing the cavity, or disinfecting a root canal, or devitalizing a pulp, or obtunding sensitive dentine, I almost invariably cover it with the oxysulphate. It is a very difficult matter to cover a pledget of cotton, well saturated with liquid, with gutta percha, particularly in a shallow cavity, but it may be very easily accomplished with oxysulphate. The necessary experience in the manipulation of the material is best acquired by making a few fillings out of the mouth. It is particularly in making applications of arsenic to the dental pulp that the manner of enclosing them has great advantages, as it admits of keeping a local anæsthetic constantly in contact with the pulp, and avoids the pressure which is too frequently a cause of severe pain following such applications.

"6. I now and then use the oxysulphate for mixing metallic caps over the teeth in regulating appliances where they are to remain but a short time, also for temporarily setting pivot teeth. In short, any one who becomes acquainted with the material will find it so useful that he will wonder how he was ever able to get along without it."—*Dominion Dental Journal.*

